# Advanced Multimodality Cardiovascular Imaging in Patients at Very High Cardiovascular Risk Without a Previous Cardiovascular Event: Current Knowledge and Future Perspectives

**DOI:** 10.3390/jcdd13060234

**Published:** 2026-05-30

**Authors:** Federica Marzano, Ermanno Nardi, Ciro Cotticelli, Mariafrancesca Di Santo, Simone Agizza, Giuseppe Maria Abbellito, Fabrizio Perrone Filardi, Laura Liccardi, Salvatrice Di Sarno, Isabel Martone, Stefania Paolillo, Paola Gargiulo, Sara Maria Pizzileo, Francesco Pizzolorusso, Andrea Igoren Guaricci, Giuseppe Guglielmi, Pasquale Perrone Filardi

**Affiliations:** 1 Department of Advanced Biomedical Sciences, University of Naples Federico II, 80131 Naples, Italy; 2 Department of Translational Medical Sciences, University of Naples Federico II, 80131 Naples, Italy; 3 Department of Clinical and Experimental Medicine, University of Foggia, 71122 Foggia, Italy; 4 University Cardiology Unit, Interdisciplinary Department of Medicine, Polyclinic University Hospital, University of Bari Aldo Moro, 70121 Bari, Italy

**Keywords:** subclinical atherosclerosis, cardiovascular risk stratification, primary prevention, multimodality imaging

## Abstract

A substantial proportion of cardiovascular (CV) events occurs in individuals without previously diagnosed CV disease, underscoring the need for improved primary prevention strategies. Traditional risk scores provide probabilistic estimates but fail to directly identify the presence and heterogeneity of subclinical atherosclerosis. This review summarizes current evidence on advanced multimodality imaging approaches for identifying high-risk individuals without prior CV events. Evidence from cohort studies, randomized trials, and meta-analyses was examined to evaluate the role of coronary artery calcium (CAC) scoring, coronary computed tomography angiography (CCTA), perivascular fat attenuation index (FAI), and vascular ultrasound in risk stratification. CAC scoring remains the most validated and widely recommended tool, offering robust prognostic value and significant risk reclassification, particularly in intermediate-risk individuals. CCTA provides additional insights into plaque burden and high-risk phenotypes, while FAI enables noninvasive assessment of coronary inflammation, improving risk prediction beyond anatomical measures. Vascular ultrasound offers a radiation-free, accessible method for detecting systemic plaque burden and refining risk estimation. Overall, multimodality imaging enhances the identification of subclinical disease and supports more individualized, disease-based risk assessment. Future research should clarify cost effectiveness, optimize patient selection, and determine whether imaging-guided strategies improve long-term clinical outcomes.

## 1. Introduction

A substantial proportion of cardiovascular (CV) events occur as first events in individuals without previously diagnosed CV disease [1]. This persistent clinical burden highlights a central challenge in primary prevention: identifying patients who are already at very high CV risk despite the absence of prior events and who may therefore benefit from earlier and more intensive preventive strategies.

Atherosclerosis typically develops silently over a highly variable latency period, during which coronary and peripheral arterial disease may already be present but remain clinically asymptomatic or subclinical [2]. Subclinical atherosclerosis is remarkably common in the general population, and its prevalence increases substantially with age, with higher rates in men compared to women [3,4]. Accordingly, the critical unmet need is to recognize subclinical atherosclerosis before the first clinical event occurs.

Contemporary risk prediction models, including SCORE2 and SCORE2-OP [5,6], represent a cornerstone for primary prevention; yet, their performance at the individual level remains limited, as they only provide an estimate of individual risk rather than directly identifying the presence of disease. Recent European Society of Cardiology (ESC)/European Atherosclerosis Society (EAS) recommendations emphasize that additional risk modifiers should be considered to refine risk classification and guide the intensity of preventive strategies [7,8]. Notably, the 2025 ESC/EAS Focused Update [9] recognizes subclinical atherosclerosis detected by imaging, including coronary artery calcium (CAC) scoring and plaque burden on coronary or peripheral arterial imaging, as relevant modifiers of CV risk.

Overall, advanced multimodality CV imaging has emerged as a valuable tool in primary prevention risk assessment, enabling the identification of subclinical atherosclerosis and better recognition of event-free individuals at higher risk [10,11].

CAC assessment via computed tomography (CT) represents a robust, reproducible marker of subclinical coronary atherosclerotic burden [12,13], whereas coronary CT angiography (CCTA) enables a more comprehensive evaluation of plaque extent and composition [14]. In parallel, perivascular fat attenuation (FAI) has emerged as a promising noninvasive marker of coronary inflammation, providing insights into plaque biology beyond traditional anatomic assessment [15]. In addition, vascular ultrasound provides a radiation-free modality to detect early atherosclerosis and prevent CV events in selected populations [16,17].

In this review, we summarize current evidence on advanced multimodality imaging for identifying very-high-risk patients without a prior CV event, discuss practical strengths and limitations of these techniques, and outline future perspectives toward imaging-guided precision prevention.

## 2. Subclinical Atherosclerosis: Beyond Traditional Risk Scores

Subclinical atherosclerosis develops over years or even decades of latency. It represents a pathophysiological continuum characterized by functional, cellular, and molecular alterations in the vascular wall that precede the formation of clinically evident lesions [18]. The presence of atherosclerotic CV disease (ASCVD) in individuals without prior acute CV events is an increasingly frequent scenario, largely driven by population aging [19]. Current European and American guidelines recommend CV risk stratification based on models derived from epidemiological cohorts integrating traditional risk factors [7,20]. However, these tools present substantial limitations, primarily related to their probabilistic nature and reliance on static variables, which do not adequately capture the complexity and heterogeneity of the atherosclerotic process [21]. Individuals with a seemingly favorable CV risk profile may present with biologically advanced subclinical ASCVD, whereas others at theoretically high risk may remain event-free for prolonged periods [22]. Accordingly, a significant proportion of CV events occur in individuals classified as low- or moderate-risk but with evidence of subclinical ASCVD, supporting the need for an approach based on early identification [22]. Another relevant aspect is the temporal dimension of CV risk. Traditional scores estimate the 10-year risk of fatal and non-fatal CV events, potentially underestimating lifetime exposure, particularly in younger individuals [21]. In this setting, the identification of early vascular damage enables a shift toward a more direct and individualized CV risk assessment. For instance, in the ARIC (Atherosclerosis Risk In Communities) study [23], the presence of carotid plaque led to a reclassification of 23% of the subjects to a higher CV risk category, including 13.5% of those initially considered at intermediate risk [23]. Consistently, studies incorporating CT-derived markers of atherosclerosis have demonstrated substantial rates of risk reclassification, with up to 40–50% of individuals reassigned to different risk categories and a significant proportion of intermediate-risk subjects reclassified to higher-risk groups [24,25]. The most recent European guidelines strongly support this approach, recommending the integration of CV risk modifiers and the detection of subclinical ASCVD to refine CV risk stratification [7,26,27]. From a clinical perspective, the incorporation of subclinical ASCVD into risk assessment has important therapeutic implications [28]. The presence of documented ASCVD places the patient into a very-high-risk category, supporting the idea of an early intensification of therapy in subjects with a high atherosclerotic burden [7,26,27]. Conversely, the absence of detectable vascular damage may help refine the CV risk and avoid overtreatment in selected individuals, improving the overall appropriateness of care [7,26,27,28].

## 3. CAC as a Validated Imaging Marker of Subclinical Coronary Atherosclerotic Burden

**Figure 1:**
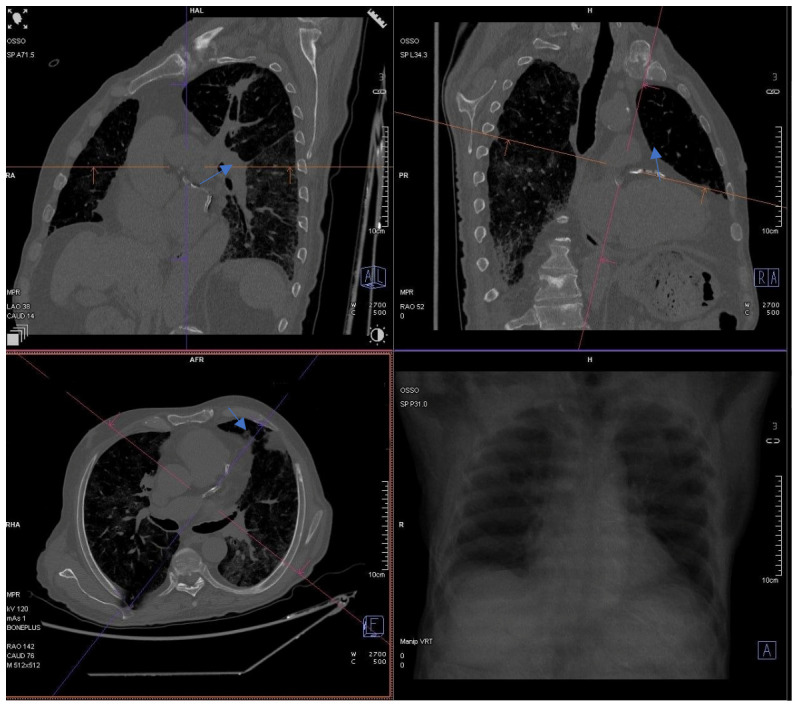
Non-contrast chest CT scans reveal calcific atherosclerosis of the LAD. The images show axial, coronal, and sagittal planes obtained from the LAD. Arrows in all panels indicate diffuse calcific atherosclerosis along the LAD.

CAC scoring is a highly robust imaging marker of subclinical coronary atherosclerotic burden, providing powerful prognostic information that is incremental to traditional CV risk factors. The most recent European guidelines recommend the CAC quantification via CT in CV risk stratification, particularly for primary prevention in asymptomatic individuals at intermediate or borderline risk [20,27]. CAC quantifies the total burden of coronary atherosclerosis through a simple, non-contrast CT scan and has been consistently validated as a predictor of CV events across diverse populations [29] (Figure 1). From a pathophysiological perspective, CAC represents the mineralization of atherosclerotic plaques, a relatively advanced stage of disease, that closely correlates with total atherosclerotic burden independent of the presence of significant coronary stenoses [30].

Quantification is typically performed using the Agatston score, which ensures high reproducibility and standardization, thus guiding decision-making and prescription of primary prevention therapies. According to the Agatston score, CAC is typically stratified into categories, ranging from 0, corresponding to an undetectable atherosclerotic plaque, to mild (1–99), moderate (100–299), high (300–999), or severe (≥1000) atherosclerotic involvement [29]. Very high CAC values (≥1000) in patients without known CV disease from Multi-Ethnic Study of Atherosclerosis (MESA) cohort have been associated with markedly elevated risk of major adverse CV events (MACE) and mortality, warranting more aggressive therapeutic strategies [31]. In addition, in two population-based cohorts of 3208 adults from the MESA and Rotterdam Study, aged 45–79 years, adding CAC to traditional risk-factor models produced a meaningful improvement in coronary heart disease (CHD) risk discrimination, with consistent results across age strata [32]. In a recent meta-analysis of 45 studies, the presence of any coronary artery calcium (CAC > 0) was associated with a 4-fold increased risk of MACE (RR 4.05; 95% CI 2.91–5.63) and a 3-fold increased risk of all-cause mortality (RR 3.23; 95% CI 2.12–4.93) compared to CAC = 0 in asymptomatic individuals without established CHD over a mean follow-up of 11 years, supporting the use of CAC scoring as a powerful risk stratification tool in primary prevention [33].

Technological advances have further expanded the clinical applications of CAC, including the implementation of artificial intelligence (AI) algorithms. For instance, in a large primary-prevention cohort of 5678 adults without established ASCVD, undergoing routine non-ECG-gated chest CT, deep learning-quantified incidental CAC provided incremental risk stratification beyond traditional risk factors; in particular, CAC ≥ 100 was independently associated with increased risk of all-cause mortality (HR 1.51; 95% CI 1.28–1.79), death/myocardial infarction (MI)/stroke (HR 1.57; 95% CI 1.33–1.84), and death/MI/stroke/revascularization (HR 1.69; 95% CI 1.45–1.98), compared to CAC = 0 [34]. More recently, in 5830 asymptomatic MESA participants followed for 15 years, an AI-derived analysis of standard CAC scans (AI-CAC) predicted overall CV events, including heart failure, atrial fibrillation, and stroke, significantly better than the Agatston score across multiple time horizons. AI plaque features also improved CHD prediction in the mild CAC (1–100) range, highlighting CAC scans as a source of prognostic information beyond Agatston alone [35].

## 4. CCTA and High-Risk Plaque Phenotypes in Primary Prevention

**Figure 2:**
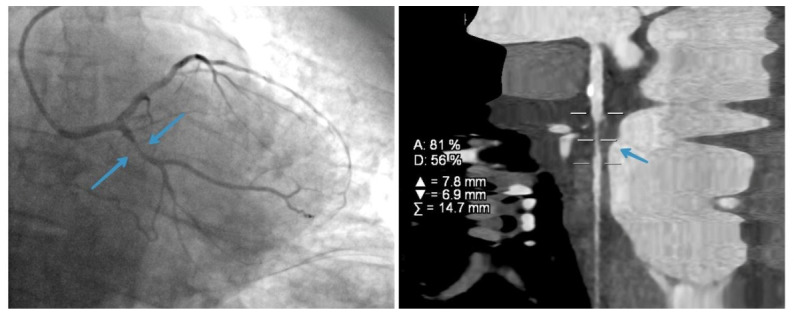
Coronary angiography (**left**) and CCTA (**right**) in hypertensive, obese, former smoker patient with episodes of retrosternal chest pain. CCTA successfully identified a concentric “soft” plaque in the proximal segment, extending for approximately 8–9 mm and causing severe stenosis of about 80% of the CX. Blue arrows indicate the site of the CX stenosis as visualized on coronary angiography (**left**) and CCTA (**right**). *(A = Area stenosis (%); D = Diameter stenosis (%); ▲ = Reference vessel diameter (mm); ▼ = Minimal lumen diameter (mm); Σ = Lesion length (mm)*.

CCTA has emerged as a superior tool for noninvasive assessment of subclinical atherosclerosis, providing detailed anatomic information beyond CAC scoring, including noncalcified plaque burden, high-risk plaque features (such as low-attenuation plaque, positive remodeling, and spotty calcifications), and degree of stenosis [36]. The use of iodinated contrast media ensures clear opacification of the coronary lumen, facilitating the identification of stenosis or anomalies (Figure 2). From a technical standpoint, coronary CT studies can be performed using various electrocardiographic synchronization methods; among these, retrospective gating is one of the most comprehensive acquisition techniques. In a secondary analysis of the PROMISE trial, performed on 4415 stable symptomatic outpatients, with a median follow-up of 25 months, high-risk plaque features on CCTA were associated with a higher incidence of MACE [37]. Moreover, in the SCOT-HEART randomized trial, including 4146 patients with stable chest pain, adding CCTA to standard care reduced the 5-year rate of the primary composite endpoint of CHD death or nonfatal MI compared with standard care alone (HR 0.59; 95% CI 0.88–1.13) [38]. CCTA led to more early invasive testing and initiation of preventive therapies, with similar rates of coronary angiography or revascularization between groups [38]. In a post hoc analysis of this trial, including 1769 patients with stable chest pain with a median follow-up of 4.7 years, noncalcified low-attenuation plaque burden on CCTA emerged as the strongest independent predictor of future fatal/nonfatal MI, irrespective of traditional risk scores, CAC, and stenosis severity [39].

In the recently published SCAPIS analysis, including 24,791 adults without CVD, followed for a median time of 7.8 years, CCTA measures of plaque extent and noncalcified disease were independently associated with incident nonfatal MI or CHD death, providing improved prediction of coronary events beyond traditional risk factors and CAC score [40].

In a recent meta-analysis pooling 75 studies, CCTA-based analyses, including CT-derived fractional flow reserve (FFR), high-risk plaque features, FAI, total plaque volume, and radiomics scores, were consistently associated with MACE. The strongest associations were observed for CT-FFR (HR 6.14; 95% CI 3.75–10.05) and high-risk plaque (HR 4.05; 95% CI 3.16–5.18), although heterogeneity across studies was moderate-to-high. Overall, the findings support advanced CCTA analyses as promising tools to refine CV risk stratification [14]. Although CCTA provides detailed characterization of coronary plaque burden and vulnerability features, its broader implementation in primary prevention remains controversial. Compared with CAC scoring, CCTA is associated with higher costs, greater radiation exposure, and the need for contrast administration. Therefore, until ongoing randomized trials clarify the net clinical benefit and cost effectiveness of CCTA-guided prevention strategies, careful patient selection remains essential, and CAC scanning remains the preferred screening strategy for asymptomatic populations [41]. In this context, the ongoing SCOT-HEART 2 (NCT03920176) and DANE-HEART (NCT05677386) trials have been designed to evaluate CCTA as a primary prevention risk assessment strategy, in order to determine whether CCTA benefits exceed those of CAC and traditional risk scores [42]. Major international guidelines, such as those from the European Society of Cardiology (ESC), now recognize CCTA as playing a leading role in the evaluation of stable coronary artery disease. Specifically, the examination is indicated as a first-line test for patients with a low-to-intermediate pre-test probability of coronary artery disease owing to its high negative predictive value. It is also applied in the evaluation of congenital coronary anomalies, the study of coronary artery bypass grafts, and, in selected cases, stent follow-up.

## 5. Imaging of Plaque Inflammation with Perivascular FAI: From Pathophysiology to Risk Prediction

FAI represents an innovative imaging biomarker that quantifies coronary inflammation through analysis of pericoronary adipose tissue (PCAT) by performing routine CCTA [43]. The underlying pathophysiology is based on bidirectional signaling between the vascular wall and surrounding perivascular adipose tissue. Vascular inflammation induces structural changes in PCAT through lipolysis and inhibition of adipogenesis, providing a reduction in lipid content of perivascular adipocytes and consequent increased CT attenuation [44,45,46].

**Figure 3:**
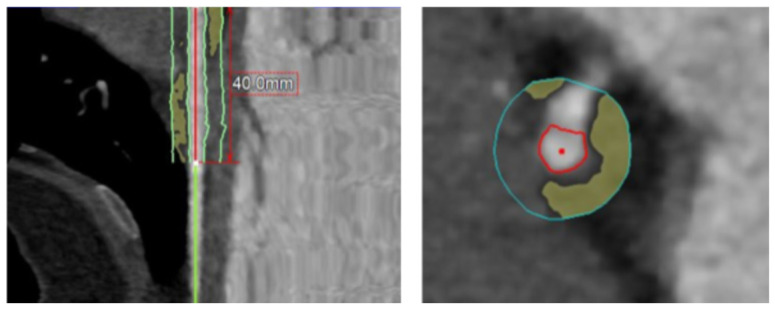
Coronary CT reconstruction of the right coronary artery. Selection of the region of interest analysis showing the distribution of values between −190 and −30 HU (yellow) in long-axis (**left**) and short-axis views (**right**). Red lines indicate centerline in long-axis and the perimeter of the coronary artery visualized during the angiographic phase in short-axis.

According to current clinical guidelines, CCTA is a sensitive and widely performed imaging modality to identify obstructive coronary artery disease, focusing on the identification of coronary artery plaques (Figure 3). From a prognostic perspective, FAI has demonstrated independent and incremental predictive value beyond traditional CV risk factors and anatomic CCTA parameters.

Routine detection and quantification of coronary artery inflammation could represent a key strategy to stratify risk in patients without anatomically significant coronary artery disease.

The milestone CRISP-CT Study identified a threshold FAI value ≥ −70.1 Hounsfield units as predictive of both all-cause mortality and cardiac mortality, with respectively HR of 2.55 (95% CI 1.65–3.92) and 9.04 (95% CI: 3.35–24.40) in the derivation cohort and 3.69 (95% CI 2.26–6.02) and 5.62 (95% CI 2.90–10.88) in the validation cohort [15].

A meta-analysis of 6335 patients confirmed that elevated pericoronary FAI is associated with a three-fold increased risk of MACE (HR 3.29; 95% CI: 1.88–5.76) [47].

In the ORFAN Study, which enrolled about 40,000 patients, over 80% of patients undergoing CCTA had no significant coronary artery stenosis and paradoxically accounted for most CV events over a median follow-up of 2.7 years. Based on findings of the ORFAN Study, elevated FAI scores across all three coronary arteries demonstrated an additive significant impact on cardiac mortality risk (HR 29.8; 95% CI 13.9–63.9) and MACE (HR 12.6; 8.5–18.6) when comparing three vessels with FAI scores in the upper versus lower quartile [48]. Moreover, the integration of FAI into AI algorithms (AI-Risk) has further enhanced risk stratification by combining coronary inflammation with plaque burden and clinical risk factors [48,49].

Clinical applications of FAI extend from vulnerable plaque identification to monitoring responses to anti-inflammatory therapies [43]. In the field of preventive medicine, FAI may play a crucial role in detecting early signs of coronary involvement before the development of “vulnerable plaques,” leading to targeted primary prevention strategies and optimization of intensive secondary prevention therapy, including anti-inflammatory agents or proprotein convertase subtilisin/kexin type-9 inhibitors in patients with elevated residual inflammatory risk [45,50]. Despite its promising prognostic performance, several limitations currently restrict the widespread clinical implementation of FAI. Measurements may vary according to scanner technology, acquisition protocols, reconstruction algorithms, and technical parameters, potentially affecting reproducibility across centers. In addition, standardized cut-off values have not yet been universally validated across different populations and clinical settings. Most available evidence derives from observational studies conducted in highly specialized centers, while the cost effectiveness and clinical impact of FAI-guided strategies remain to be established [43,47].

## 6. Vascular Ultrasound for Subclinical Atherosclerosis Detection

**Figure 4:**
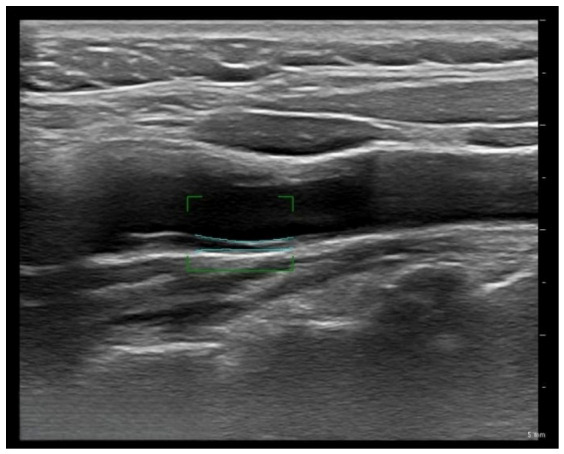
Example of a carotid ultrasound showing an IMT value at the upper limits of normal (0.96 mm), in a patient with CV risk factors, supporting the role of vascular ultrasound in the assessment of subclinical vascular damage. The green box and the blue line indicate the site where the IMT measurement was performed.

Vascular ultrasound may provide valuable complementary information by enabling direct visualization of arterial wall abnormalities and plaque burden, thereby identifying patients in whom the true atherosclerotic burden may be underestimated by conventional risk algorithms (Figure 4). In line with this concept, post-guideline evidence has shown that SCORE2 may overestimate risk in individuals without carotid plaque (3.93% observed vs. 5.89% predicted) and underestimate risk in those with plaque (9.69% observed vs. 8.12% predicted), while the inclusion of carotid plaque significantly improves predictive performance [51]. These findings support the use of ultrasound-based markers of subclinical atherosclerosis to refine traditional risk estimation. Consistently, the 2019 ESC/EAS dyslipidaemia guidelines recommended carotid and/or femoral plaque assessment as a CV risk modifier in asymptomatic individuals at low-to-moderate risk, with a Class IIa recommendation and Level of Evidence B, an approach maintained in the 2025 focused update [8,9].

Beyond its role in risk reclassification, vascular ultrasound is non-invasive, widely available, radiation-free, and relatively inexpensive, making it attractive for large-scale primary prevention.

In the pragmatic VIPVIZA randomized trial, including 3532 individuals, carotid ultrasound screening paired with a pictorial report of subclinical atherosclerosis (shared with patients and primary care physicians and reinforced by a nurse call) resulted in more favorable 1-year CV risk profiles compared to usual care, supporting ultrasound-based communication as a tool to enhance primary prevention effectiveness [52].

Importantly, not all ultrasound-derived markers carry the same clinical value. While carotid intimal medial thickness (IMT) has historically been regarded as an indicator of early vascular damage, current evidence suggests that focal plaque more accurately reflects true atherosclerotic burden and future CV risk [51,53,54]. Indeed, in a primary-prevention clinical cohort of 1569 patients without known CV disease, carotid IMT and plaque presence alone were not independently associated with incident composite CV disease [55]. In contrast, having three or more carotid bifurcation plaques compared to no plaques predicted higher event risk (HR 2.9; 95% CI 1.48–5.65), even after adjustment for standard risk scores [55]. This study supports carotid ultrasound as a practical risk stratification tool when it quantifies plaque number/burden rather than relying on IMT alone. Moreover, vascular ultrasound should not be confined to the carotid district. Peripheral arterial imaging, particularly ultrasound of the femoral arteries, may further improve the detection of systemic subclinical atherosclerosis, and a combined carotid–femoral approach may offer a more comprehensive assessment of plaque burden [56]. In this context, a recent prospective cohort study conducted in 985 adults without followed for about 13 years, demonstrated that adding ultrasound plaque measures improved ASCVD event prediction beyond traditional risk factors [16]. Risk prediction was strongest when plaque burden was assessed at both carotid and femoral bifurcations (multisite assessment) rather than at a single site [16]. Nonetheless, operator dependency, heterogeneity of acquisition protocols, and lack of standardization still limit broader implementation. Future studies should clarify the most clinically relevant ultrasound parameters and define the patient subsets most likely to benefit from ultrasound-based risk refinement.

## 7. Discussion

Despite major advances in CV disease prevention, substantial geographical and inter-individual disparities persist, and the burden of disease remains high across many populations. Accordingly, one of the key challenges in contemporary cardiology is not only to treat overt CV disease, but also to identify high-risk individuals during the preclinical phase, when preventive strategies may be most effective. In view of the major social and economic impact of ASCVD, increasing attention has been directed toward improving primary prevention, as reflected in the 2021 ESC Guidelines on CV disease prevention. A major innovation of these guidelines was the incorporation of apparently healthy individuals into structured risk assessment pathways through age-specific tools such as SCORE2 and SCORE2-OP, which estimate the 10-year risk of fatal and non-fatal CV events in individuals without established atherosclerotic CV disease [7]. However, although these models integrate major clinical risk factors, they do not directly account for imaging evidence of subclinical atherosclerosis. Multimodality imaging in primary prevention combines CAC scoring, vascular ultrasound, and CCTA to detect subclinical atherosclerosis and refine CV risk stratification. CAC scoring has the strongest evidence base and is the most widely recommended imaging modality for intermediate-risk patients, though emerging data support complementary roles for carotid/femoral ultrasound and CCTA in selected populations. In this context, the PESA (Progression of Early Subclinical Atherosclerosis) Study, conducted on a prospective cohort of 4.184 asymptomatic middle-aged participants, integrated multimodality imaging, including multi-territory ultrasound and CAC scoring, to detect early atherosclerosis burden [57]. The results from this study have shown that 63% of participants had evidence of subclinical atherosclerosis, whereas CAC was present in only 18%. However, approximately 60% of participants without coronary calcium had plaques at other vascular sites, demonstrating that a CAC score of zero does not exclude atherosclerosis [57]. Importantly, 40% of participants classified as low long-term risk and 60% of those at intermediate risk by traditional risk equations had evidence of atherosclerosis, demonstrating that traditional risk scores miss substantial atherosclerotic burden [57]. By visualizing subclinical atherosclerosis, advanced CV multimodality imaging enables disease-based risk phenotyping beyond probabilistic scores, especially in patients close to treatment decision thresholds, in whom risk reclassification has the highest potential clinical impact [17]. However, implementation of imaging techniques in primary prevention requires a balance between diagnostic yield, cost, availability, and safety. From a practical perspective, multimodality imaging should not be viewed as the indiscriminate combination of multiple techniques, as each imaging modality provides distinct strengths and limitations, and its use should be tailored according to the specific clinical question, patient profile, and resources available. For instance, in low to intermediate CV risk asymptomatic individuals, CAC scoring provides an efficient approach to quantify coronary atherosclerotic burden, particularly when treatment decisions remain uncertain after conventional risk estimation. In contrast, according to current guidelines, CCTA may be more appropriate in individuals with low-to-moderate pre-test likelihood of obstructive coronary artery disease and could be reserved for selected patients in whom detailed plaque characterization could influence therapeutic management [27]. However, higher costs and radiation exposure limit its utilization for broad screening in asymptomatic populations [39,40]. Emerging techniques may further refine risk prediction, particularly among individuals with nonobstructive disease in whom CV risk is often underestimated [43,44]. In this context, FAI may provide incremental information regarding residual inflammatory risk and plaque vulnerability, although its clinical implementation currently remains limited by insufficient standardization and availability. Vascular ultrasound may represent a complementary radiation-free approach for systemic plaque assessment, particularly in younger individuals or settings where CAC scoring or CCTA are less accessible. Therefore, the clinical value of multimodality imaging likely resides not in replacing traditional risk scores but in refining individualized risk estimation and guiding personalized preventive strategies.

## 8. Conclusions and Future Directions

Advanced multimodality CV imaging can identify subclinical atherosclerosis and refine CV risk stratification in event-free patients who might be misclassified by traditional risk algorithms. Together, CAC scoring, vascular ultrasound, CCTA, and emerging biomarkers such as perivascular FAI provide complementary information on atherosclerotic burden, plaque phenotype, and inflammatory activity, enabling a more comprehensive imaging-based risk assessment beyond traditional algorithms. Despite these advances, further randomized trials are needed to clarify whether a multimodality imaging approach improves CV outcomes and is cost-effective compared to conventional risk-score approach. Broader implementation through standardized acquisition protocols, consistent plaque definition, and harmonized reporting formats is also required to improve reproducibility and facilitate the integration of multimodality imaging into clinical decision tools. Additionally, the introduction of AI algorithms may reduce operator dependence by automating plaque detection and phenotyping, and quantification of the plaque burden. Future research should prioritize the identification of specific subgroups of patients most likely to benefit from the use of each unique imaging technique and the development of integrated prediction models, including both clinical variables and imaging-related markers of atherosclerotic burden, thus ensuring that improved risk detection translates into durable clinical benefit.

## Data Availability

No new data were created or analyzed in this study.

## Abbreviations

The following abbreviations are used in this manuscript:

AI	Artificial intelligence
ASCVD	Atherosclerotic cardiovascular disease
CHD	Coronary heart disease
CV	Cardiovascular
CT	Computed tomography
CAC	Coronary artery calcium
CCTA	Coronary computed tomography angiography
EAS	European Atherosclerosis Society
ESC	European Society of Cardiology
FAI	Fat attenuation index
FFR	Fractional flow reserve
IMT	Intimal medial thickness
MACE	Major adverse cardiovascular events
MESA	Multi-Ethnic Study of Atherosclerosis
MI	Myocardial infarction
PESA	Progression of Early Subclinical Atherosclerosis
PCAT	Pericoronary adipose tissue
